# Copenhagen Mesenteric stent study (COMESS)—a randomized trial of stent versus covered stent treatment for chronic mesenteric ischemia

**DOI:** 10.1186/s13063-024-08285-5

**Published:** 2024-06-28

**Authors:** Alexandra A. Brandtzäg, Jonas P. Eiberg, Lars Lönn, Mikkel Taudorf, Timothy A. Resch

**Affiliations:** 1 Department of Vascular Surgery, Heart Center, Copenhagen University Hospital - Rigshospitalet, Copenhagen, Denmark; 2 Department of Clinical Medicine, University of Copenhagen, Copenhagen, Denmark; 3 Department of Diagnostic Radiology, Center of Diagnostic Investigation, Copenhagen University Hospital - Rigshospitalet, Copenhagen, Denmark

**Keywords:** Mesenteric ischemia, Stent, Endovascular treatment, Randomized controlled trial

## Abstract

**Background:**

Current management of mesenteric ischemia is primarily endovascular stent treatment. Typical CMI symptoms are postprandial abdominal pain, food fear, weight loss, and diarrhea. Revascularization is often necessary, as mesenteric ischemia may progress to bowel necrosis and death if left untreated. This study aims to compare the outcome using bare metal stent (BMS) or covered stent (CS) in the endovascular treatment of chronic and acute on chronic mesenteric ischemia.

**Methods:**

This is an investigator-driven, prospective, randomized, single-blinded, and single-center, national cohort study at the Copenhagen University Hospital, Denmark. A total of 98 patients with chronic mesenteric ischemia (CMI) and acute-on-chronic mesenteric ischemia (AoCMI) will be randomized to treatment with either BeSmooth BMS (Bentley Innomed GmbH) or BeGraft CS (Bentley Innomed GmbH). Randomization occurs intraoperatively after lesion crossing.

**Discussion:**

There is currently no published data from prospective controlled trials regarding the preferred type of stent used for the treatment of chronic and acute-on-chronic mesenteric ischemia. This trial will evaluate the short- and long-term outcome of BMS versus CS when treating CMI and AoCMI, as well as the benefit of a more intense postoperative surveillance program.

**Trial registration:**

ClinicalTrials.gov NCT05244629. Registered on February 8, 2022.

## Introduction

### Background and rationale {6a}

Chronic mesenteric ischemia (CMI) is predominantly caused by atherosclerotic [1], ostial lesions in one of the three main, mesenteric arteries: celiac trunk (CA), superior mesenteric artery (SMA), and inferior mesenteric artery (IMA). These lesions are often associated with other manifestations of atherosclerotic disease [2].

Symptomatic CMI accounts for less than 1 per 100,000 admissions; the observed rise in reported cases of symptomatic CMI aligns with the increased prevalence and precision of imaging technologies [1, 3, 4]. Atherosclerosis is the most common cause, and in most patients, the development of symptomatic CMI may take months or years to become clinically apparent. In patients with known atherosclerotic disease, the prevalence ranges from 8 to 70% and a > 50% stenosis of more than one mesenteric artery is detected in up to 15% of cases. In patients with peripheral artery disease, significant stenosis or occlusion of at least one mesenteric artery is found in 40% and 25–29%, respectively [1].

Atherosclerosis is a chronic inflammatory disease. Besides classic risk factors (cholesterol levels, smoking, high blood pressure, diabetes, and hereditary conditions), a significant coincidence between inflammatory mechanisms and symptomatic manifestation of cardiovascular disease may exist. For example, it has been shown that an increased concentration of inflammatory markers in the blood (i.e., CRP) is associated with an increased risk of atherosclerosis supporting the hypothesis that inflammatory mechanisms are essential in the development of atherosclerosis. Identification of peptides and protein from the Copenhagen Mesenteric stent study (COMESS) patients will be performed on the basis of collected data as well as in silico-generated libraries.

In this project on mesenteric ischemia, we have used a definition based on the clinical presentation: CMI, acute mesenteric ischemia (AMI), and acute on chronic mesenteric ischemia (AoCMI). CMI is defined as ischemic symptoms caused by insufficient blood supply to the gastrointestinal tract for at least 3 months. The typical presentation includes postprandial pain (88.2%), weight loss (79.2%) [5], and/or unexplained diarrhea (56%) [6]. AoCMI is defined as AMI in patients with a history of CMI. Often, the symptoms of CMI accelerate with periods of prolonged and intensified pain, pain even without eating, the onset of diarrhea, or inability to eat at all.

The abundant arterial collateral circulation of the mesenteric tract often prevents gastrointestinal ischemia in single-vessel disease. Stenosis in a single mesenteric artery is quite prevalent in the general population (up to 18%), but the diagnosis of mesenteric ischemia is uncommon partly because symptomatic CMI is rare unless two of the three mesenteric arteries are significantly stenosed or occluded [1, 7]. An exception is isolated, significant stenosis or occlusion of the SMA. This is sometimes associated with clinically relevant mesenteric ischemia as the significance of SMA flow to the intestines is particularly relevant [1].

Revascularization is indicated in patients who develop symptomatic CMI. There is no role for a conservative approach with long-term chronic parenteral nutrition and non-interventional therapy. Excessive delays in proceeding with definitive revascularization, or use of parenteral nutrition alone, have been associated with clinical deterioration, bowel infarction, and risk of sepsis from catheter-related complications [1]. The goals of mesenteric revascularization include relief of symptoms, improving quality of life, restoration of normal weight, and preventing bowel infarction [5].

Revascularization strategies to treat CMI continue to evolve with the rapid development of novel endovascular devices and techniques. During the last decade, the number of mesenteric arterial revascularizations has increased tenfold because of increasing recognition and the advent of endovascular therapy, which allows a less invasive treatment alternative. In most centers, angioplasty and stenting have become the primary treatment modalities, relegating open surgical bypass to patients who are not candidates or fail endovascular therapy [5]. The SMA is the primary target for revascularization whenever possible. Revascularization of the CA or IMA can also be performed, particularly when the SMA cannot be revascularized.

According to the guidelines from the European Society of Vascular Surgery (ESVS) as well as the US Society of Vascular Surgery (SVS), routine mesenteric stenting should be used as opposed to plain balloon angioplasty (POBA) [1, 6, 8]. In this setting, the ESVS state that CS (covered stent), as opposed to BMS (bare metal stent), may be considered, whereas the SVS recommend using CS primarily. BMS tends to form intimal hyperplasia, which is well described in the common iliac arteries, where covered stents seem to have superior patency compared to BMS. Similar tendencies are known in the setting of fenestrated aortic stent-grafting for aortic aneurysms, where CS in the renal arteries have better outcomes than BMS. The advantage of BMS is that they often require smaller access sheaths for delivery, they come on various size wire platforms, there are a large number of product types available on the market, and they are less expensive. CS sometimes require slightly larger access sheaths, are primarily delivered on a 0.035 platform, there are limited number of options commercially available, and they are more expensive.

In the current trial, CMI and AoCMI subjects will be randomized to treatment with either a BMS (BeSmooth, Bentley Innomed GmbH) or CS (BeGraft, Bentley Innomed GmbH). The metal component of the stent is identical in both groups, and the only difference is the graft covering the outside of the stent. This minimizes bias in outcomes that might be based on differing properties in the stent itself instead of the graft covering. Both the CS and the BMS are indicated and CE marked for iliac and renal stenting.

Patients with AMI will not be randomized, as this patient group is very diverse and with significant acute mortality thus preventing stable analysis of long-term stent behavior and outcome. Patients with AMI will in parallel be prospectively enrolled in a longitudinal, non-randomized cohort study according to current standard treatment.

### Objectives {7}

To assess outcomes in chronic mesenteric ischemia due to atherosclerosis, comparing the effectiveness of BMS with CS in preventing vessel re-stenosis at 12 months, and concurrently, investigate the potential advantages of an intensified postoperative surveillance program beyond current guidelines. Additionally, aim to identify inflammation-associated proteins and signaling pathways within the arteriosclerotic region of the mesenteric artery.

### Trial design {8}

This is a single-blinded, single-center, national, prospective, randomized controlled trial comparing covered stents and bare metal stents in the treatment of mesenteric vessel stenosis in patients with CMI. The COMESS trial is a superiority trial, with two parallel groups and a primary end point of primary stent patency at 1 year. Randomization is done as block randomization with a 1:1 allocation ratio.

## Methods: participants, interventions, and outcomes

### Study setting {9}

As the Copenhagen University Hospital, Denmark (Rigshospitalet) is the national referral center for patients with mesenteric ischemia, the COMESS study is a single-center trial of a national cohort. The COMESS trial is conducted at the Department of Vascular Surgery and the Department of Interventional Radiology, Copenhagen University Hospital, Denmark.

### Eligibility criteria {10}

#### Inclusion criteria

- Patients with symptomatic CMI of atherosclerotic or atherothrombotic etiology
- Intended endovascular treatment
- Symptoms consistent with CMI (pain, weight loss, diarrhea)
- Significant ostial stenosis (> 50%) of the superior mesenteric artery on CTA
- Significant stenosis on angiography (> 50% or > 15 mmHg pressure gradient)
- Patients > 18 years

#### Exclusion criteria

- No informed consent
- Non-atherosclerotic cause of mesenteric ischemia
- Acute mesenteric ischemia (AMI)
- Signs of acute bowel ischemia, peritonitis, laparotomy, sepsis
- Previous stent treatment in the superior mesenteric artery(ies)
- Target artery lesions > 4 cm in length
- Unable to cross lesion with guidewire
- Non-significant stenosis angiographically
- Pregnancy
- Allergies to contrast media or stent materials

### Who will take informed consent? {26a}

Patients will be asked to provide informed consent by the project responsible physician or project nurse prior to surgery, in accordance with local and national guidelines upon providing consent, each participant will receive a copy of the informed consent document for their records.

Patients with CMI and AoCMI have variable clinical presentation but are in most cases investigated and treated within 1 week of referral. Thus, it is difficult to give a standardized inform consent timeline for the study cohort. Some will receive treatment the next morning, others the next week. Subjects are recruited from patients referred for treatment of CMI/AoCMI at the Department of Vascular Surgery, Rigshospitalet.

If, during the screening of referral data by responsible physician and/or nurse, the patient is found eligible for study inclusion, the study nurse/coordinator is contacted (scenario 1). The first contact with the patient will then be by letter or via secure email to inquire if the patient wants more information regarding the trial. If affirmative, participants receive an information letter including study information (including subjects’ rights in a health science research project) and a written consent form to be signed if the subject decides to participate in the trial. The patient is offered a further information meeting and can choose to have this by telephone or physically. The patient is allowed to have a resource person (e.g., family member, friend, or acquaintance) present during this meeting. During this meeting, the patient is informed about the project verbally as well and allowed to ask questions.

Alternatively, due to a subacute scenario (scenario 2), some AoCMI patients are approached by the clinically responsible physician and/or nurse following the first physical contact with the department to inquire if the patient wants more information regarding the trial. If affirmative, participants are handed over the information letter including study information (including subjects’ rights in a health science research project) and a written consent form to be signed if the subject decides to participate in the trial. The patient is then provided a meeting with the study nurse/physician and informed verbally as well and allowed to ask questions. The patient is allowed to have a resource person (e.g., family member, friend, or acquaintance) present during this meeting. If the patient in scenario 2, due to subacute illness, cannot provide informed consent, the patient shall be considered ineligible for the study.

The description of the research project will be given verbally in layman terms and the patient can ask questions. This meeting will take place in the outpatient clinic or department in a quiet room. The verbal information will be accompanied by a briefing of the previously provided written information describing:

- Risks, benefits, complications, and unpredictable risks and events to participating
- Alternative treatment options
- That patient data, including confidential and private, is protected but may be accessed by health care personnel involved in the study
- The right to withdraw at any time without supplying a reason
- That decline to participate will be universally accepted and that the patient will receive standard care

The patient will then be given a minimum of 24 h being asked again regarding participation. If the patient accepts inclusion, the informed consent is signed. In the subacute scenario (scenario 2), a minimum of 12 h will be provided before the patient is asked again regarding participation. This reduced time is to avoid unnecessary treatment delay for the patient in a subacute clinical scenario. Patients who decline participation or are not eligible for inclusion are registered in a prospective screening log.

If new information regarding effects, risks, and side effects is discovered after inclusion or during study follow-up, participants will be promptly informed. In case of study termination, participants will be informed about the cause of termination.

### Additional consent provisions for collection and use of participant data and biological specimens {26b}

The participants in the trial will provide separate consent for the storage of residual material in a biobank for future research.

## Interventions

### Explanation for the choice of comparators {6b}

The evidence for stent treatment of CMI and AoCMI is convincing, yet there are no stents that are primarily indicated for the use in the mesenteric circulation [4, 7]. Currently used stents are indicated for renal or iliac use. Furthermore, the use of covered stents (CS) for CMI has increased, partly due to recommendations from vascular societies and extrapolation from their use in other vascular beds [1, 6]. Thus, a wide variation of stents are used, and the recent literature on BMS versus CS for arteriosclerotic occlusive disease of the superior mesenteric artery are conflicting [4, 9].

### Intervention description {11a}

The procedure will take place in an angiography suite under local anesthesia. Access will primarily be gained from the femoral artery using a 6–8 Fr sheath. An alternate vascular access route, primarily the brachial artery, can be chosen at the operator’s discretion. After the target lesion has been crossed with a 0.035 guidewire, a pressure gradient across the lesion will be determined using a mean pressure gradient > 15 mmHg to define significant lesions [5]. In the case of an occlusion or near occlusion of the SMA, intra-arterial pressure measurement is omitted as it is not technically measurable. The lesion is then treated based on the pre- and intraoperative imaging. Patients with a significant lesion will then be randomized to either BMS or CS treatment. The treating physician will choose the appropriate stent length and diameter based on pre- and intraoperative imaging. The stent should cover the entire primary lesion and protrude 3–5 mm into the aorta to achieve total lesion coverage. Bare metal stent extensions (either balloon expandable or self-expandable) can be placed at the discretion of the operator to allow optimal conformity to the target vessel or minimize the risk of target vessel dissections. The stent is dilated to minimum the nominal pressure given by the manufacturer to achieve nominal stent size.

The treatment result is evaluated by standard angiography in two planes and measuring the pressure gradient across the stented segment. Two plane angiography can be substituted by on-table beam CT or angiography in one plane combined with a single X-ray in two planes to determine stent compression. Less than 30% residual stenosis and/or mean pressure gradient < 15 mmHg is considered a treatment success.

All patients will have the balloon catheter used for ballooning the stenosis/occlusion collected. Residual material from the PTA balloon and blood samples from the angioplasty catheter (< 50 ml blood) will be collected during the procedure and stored at − 80 °C in a research biobank until analysis. Peripheral blood samples (< 50 ml blood in total) will be collected in connection with PTA treatment, as well as during the yearly outpatient visits (< 50 ml) to analyze circulating blood markers.

### Follow-up

In this trial, patients will attend outpatient visits with clinical exams, blood samples, Duplex ultrasound (DUS), and quality of life (QoL) questionnaire at 6 weeks, 12 months, and then annually to 5 years postoperatively. CTA will be performed once at 12 months postoperatively, prior to the scheduled follow-up. In case of prohibitive traveling distance to the study site, the follow-up DUS at 1 year and beyond can be omitted from the protocol and replaced by CTA at investigators discretion. As the primary study endpoint is at 1 year, this alteration will not interfere with the conclusions. If there is suspicion of an in stent re-stenosis based on the clinical exam and DUS of the SMA (threshold 412 cm/s) [6], a patient will undergo a CTA. Following evaluation of CTA results and clinical examination, the patient may be offered additional treatment if indicated.

### Criteria for discontinuing or modifying allocated interventions {11b}

As randomization takes place during the procedure, the patients can accept inclusion but be excluded during the proposed intervention if the identified arterial lesion is not confirmed to be significant by intraoperative pressure measurement or multiplane angiography. If a patient is excluded perioperatively, the operator will make a determination on whether the procedure continues according to standard treatment or if an alternative approach is deemed necessary.

The patient has the right to withdraw participation at any time without providing a reason. In the event of refusal or withdrawal, the patient will be transitioned to the standard care pathway for treatment.

### Strategies to improve adherence to interventions {11c}

All patients included in the COMESS trial will be followed up with clinical exam, blood samples, DUS of the visceral arteries, and QoL questionnaire at 6 weeks, 12 months, and then annually to 5 years postoperatively. During this time, information from exams, tests, and information will be gathered. CTA is performed at 12 months and will be done at the local hospital.

### Relevant concomitant care permitted or prohibited during the trial {11d}

There is currently no concomitant care that is prohibited during the trial. At the point of discharge from the hospital, patients will be prescribed statins and relevant antiplatelet therapy, already on other pertinent anticoagulant therapy.

### Provisions for post-trial care {30}

Patients are covered by general patient insurance (patienterstatningen).

## Outcomes {12}

### Primary endpoint

- Primary stent patency at 1 year (< 50% stenosis)

### Secondary endpoint(s)—evaluated at 12 months and 5 years

- Secondary stent patency
- Freedom from stent re-stenosis (luminal diameter > 50%)
- Freedom from reintervention
- Overall survival
- Intervention free survival
- Freedom from symptom recurrence
- Proteomics assay signal (balloon) and concentration of established biomarkers (blood samples)
- Quality of life
- Sensitivity and specificity of CTA and US preoperatively and during follow-up

### Participant timeline {13}

See Tables 1 and 2.

**Table 1:** Standard participant timeline

Enrolment	Baseline	6-week follow-up	12-month follow-up	2-year follow-up	3-year follow-up	4-year follow-up	5-year follow-up
Informed consent	X						
EQ-5D-5L	X	X	X	X	X	X	X
SF-36	X	X	X	X	X	X	X
Medical history	X						
Physical examination	X	X	X	X	X	X	X
CT-angiography	X		X				
Duplex ultrasound	X	X	X	X	X	X	X

**Table 2:** Modified participant timeline

Enrolment	Baseline	6-week follow-up	12-month follow-up	2-year follow-up	3-year follow-up	4-year follow-up	5-year follow-up
Informed consent	X						
EQ-5D-5L	X	X	X	X	X	X	X
SF-36	X	X	X	X	X	X	X
Medical history	X						
Physical examination	X	X					
CT-angiography	X		X				
Duplex ultrasound	X	X					
Telephone consultation		X	X	X	X	X	X

### Sample size {14}

In the only study investigating CS vs BMS in CMI [10], a retrospective non-randomized study of 225 patients, patients treated by CS had freedom from restenosis of 92% ± 6% compared to BMS with freedom restenosis of 53% ± 4% [10]. This study suffered several limitations that must be considered for a sample size estimation in the present RCT: the velocity cut-off used may overestimate high-grade restenosis, the follow-up was significantly longer in the BMS group, and differences in time-dependent outcomes including restenosis must be expected. In addition, a general underreporting of outcomes in both the BMS and the CS arm must be expected in a retrospective study. Therefore, the expected restenosis rates were adjusted to 85% and 60%.

Using dichotomous endpoints (restenosis: Y/N), a two independent sample study, alpha = 0.05, beta = 0.2, and power = 0.8, sample size was estimated to 49 patients in each group (total = 98). With 120 CMI/AoCMI patients treated yearly in Copenhagen, an estimated inclusion rate of 80% and 20% dropout, a total of 77 CMI patients will be randomized yearly (app 6 pts/month)—and 98 patients are expected included within 18–24 months.

### Recruitment {15}

Patients are discussed in a multidisciplinary team (MDT) at Copenhagen University Hospital (Rigshospitalet) in Denmark, including vascular surgeons and interventional radiologists after the referring physician has ruled out other gastrointestinal causes. If a consensus decision of CMI or AoCMI is reached and the diagnostic workup and clinical findings support endovascular treatment, the patient is invited to participate in the study.

## Assignment of interventions: allocation

### Sequence generation {16a}

The process of randomization is done in Research Electronic Data Capture (REDCap). Randomization will be in block sequence to get equal patient allocation in the two treatment arms in REDCap, a third party electronic dataset system, and accessible only to the physician/research nurse/coordinator performing the randomization. Only the treating team will know the allocation (CS vs BMS).

### Concealment mechanism {16b}

Randomization is done in REDCap, a password secured third party database, and is only accessible to those whom have been granted access to the project.

### Implementation {16c}

Eligible patients and the eventual next of kin will have oral and written information about the project by a project responsible physician or project nurse. As noted previously, randomization is done in Research Electronic Data Capture (REDCap), after the patient has provided informed consent. Randomization will be performed on an intention-to-stent basis in the angiosuite once the target lesion has been crossed with a guidewire and the lesion is determined clinically significant by angiography (> 50%) or pressure measurement (> 15 mmHg mean gradient). Only the treating team will know the allocation (CS vs BMS).

## Assignment of interventions: blinding

### Who will be blinded {17a}

This is a single-blinded study. Patients and the physicians responsible for postoperative care and follow-up will be blinded to treatment allocation, until the process of unblinding.

### Procedure for unblinding if needed {17b}

The unblinding process occurs at the 12-month follow-up if the patient wishes to be informed. However, in the case of severe adverse events necessitating unblinding before the 12-month follow-up, the project nurse, with access to the patient’s treatment allocation, will facilitate the unblinding process.

## Data collection and management

### Plans for assessment and collection of outcomes {18a}

Personal data concerning the subjects is protected in accordance with the Data Protection Regulation (GDPR—General Data Protection Regulation) and the Data Protection Act as well as the Health Act. Documentation for the research project is submitted to the Capital Region of Denmark’s Research List.

Data is stored and analyzed on a secure network drive with secure access only to staff directly involved in the trial conduct in pseudo-anonymized form in accordance with guidelines specified by the Danish Data Protection Agency and the GDPR. Standard blood samples for immediate analysis when monitoring treatment are labeled with subjects’ names and CPR numbers, and blood sample responses will appear in the patient record. Other research blood samples for the research biobank are pseudonymized with identification labels.

Research results belong to the trial site, and results are not handed out. The protocol is performed according to the Declaration of Helsinki II, and trial participants may withdraw from the trial at any time without further explanation.

### Plans to promote participant retention and complete follow-up {18b}

According to the existing standard of care (SOC), follow-up after mesenteric stenting at Rigshospitalet is an outpatient visit, including clinical examination and DUS at 6 weeks and 12 months postoperatively. According to guidelines, FU after stenting of mesenteric arteries includes clinical examination and DUS at 1 month, 6 months, 12 months, and annually thereafter [9].

In this trial, annual FU outpatient visits until the fifth postoperative year, including DUS, are planned. In addition, a CTA at 12 months has been added to validate the DUS findings. In summary, because of the present study, the participating patient will receive care in better adherence to internationally accepted guidelines using of CS instead of BMS (half of the patients) and yearly FU for 5 years instead of 1 year FU only (all patients).

If a patient chooses to not participate in the trial after randomization, patients will receive care according to current SOC.

### Data management {19}

Personal data concerning the subjects is protected in accordance with the Data Protection Regulation (GDPR—General Data Protection Regulation) and the Data Protection Act as well as the Health Act. Documentation for the research project is submitted to the Capital Region of Denmark’s Research List. The project responsible physician and project nurse are responsible for data management and electronic data entry of CRF in REDCap. Any paper-based CRF are stored in a secure place in the trial office.

### Confidentiality {27}

Data is stored and analyzed on a computer in pseudo-anonymized form in accordance with guidelines specified by the Danish Data Protection Agency and the GDPR. Control blood samples for immediate analysis when monitoring treatment are labeled with subjects’ names and CPR numbers, and blood sample responses will appear in the patient record. Other research blood samples for the research biobank are pseudonymized with identification labels. Data and participant details are stored on Research Electronic Data Capture (REDCap) and will only be accessible by trial members. Any paper-based CRF, including signed informed consent, are stored securely in the trial office.

### Plans for collection, laboratory evaluation, and storage of biological specimens for genetic or molecular analysis in this trial/future use {33}

The blood sample and tissue residues will constitute a research biobank located at Rigshospitalet, which will be stored for the project’s duration until the final analysis plan is available in collaboration with relevant partners at Rigshospitalet. The COMESS research biobank will be discontinued on 01–06-2031. After completing the study, the remaining material is transferred for storage in a biobank for future research, which is reported to the Capital Region’s Knowledge Centre for Data Processing and follows the Data Protection Ordinance and the Data Protection Act. The biobank for future research will be established to carry out future supplementary studies and utilize any technological development after renewed approval from relevant authorities. The participants in the experiment give separate consent for the storage of residual material in a biobank for future research.

## Statistical methods

### Statistical methods for primary and secondary outcomes {20a}

Outcomes are analyzed on intention to treat basis and secondarily per protocol. Primary outcomes will be analyzed by *t*-test and overall survival analysis, to estimate the patency at 12 months.

Secondary endpoints will be analyzed by survival analysis techniques, such as Kaplan–Meier curve and log-rank tests. ANOVA repeated measures will be used to estimate comparison QoL between the two groups, and secondary stent patency. Log-rank test will be used to compare the survival rates between groups. Sensitivity and specificity of CTA and US will be analyzed with a logistic regression and receiver operator curves (ROC) analysis.

### Interim analysis {21b}

An interim analysis will be performed in this trial by the trial steering committee after inclusion of 1/3 and 2/3 of subjects to validate safety and efficacy of the trial.

### Methods for additional analyses (e.g., subgroup analyses) {20b}

Not applicable. Subgroup analysis or any other additional analysis is not planned for this trial, as it will likely result in an insufficient sample size.

### Methods in analysis to handle protocol non-adherence and any statistical methods to handle missing data {20c}

Patients whom are not included in the trial for any reason are kept in a pseudonymized screening log to allow further analysis. Randomization of patients is performed on an intention to stent basis. Analysis of randomized patients will be done on an intention-to-treat analysis and per-protocol analysis will also be performed. To handle missing data, mixed models without imputation will be performed.

### Plans to give access to the full protocol, participant-level data, and statistical code {31c}

All results will be published, positive or negative, and potential funding bodies will have no impact on which results are published. For publication, all data is consolidated, anonymized and individual patients and outcomes not traceable. The corresponding author may provide the full protocol, statistical study and analyzed datasets, after the trial steering committee has reviewed said request.

## Oversight and monitoring

### Composition of the coordinating center and trial steering committee {5d}

The trial steering committee has been formed with representatives from the two clinical departments involved. The TSC maintains the overall responsibility and conduct of the trial. The TSC will report annually on recruitment and monitor the recording of data and violations of the protocol. They will also oversee serious adverse events, safety, and study viability.

### Composition of the data monitoring committee, its role and reporting structure {21a}

The TSC will be responsible for data monitoring activities. Ten percent of entered data will be monitored for completeness and accuracy. The TSC will meet at least bi-monthly and minutes are archived. Annual meetings with audit of internal monitoring activities and adverse events will take place and result in a recommendation to continue, terminate, or modify the trial. Data monitoring is primarily performed by the TSC.

### Adverse event reporting and harms {22}

Adverse events will be reported in REDCap, in addition to adverse events being reported according to the local guidelines. All adverse events will be adjudicated internally on a continuing basis by the study trial steering committee.

#### Harms

The risk for procedure-related complications related to the use of BMS and CS is, according to a recent observational cohort study, less than 5% and expected to be similar in both arms [5]. Most short-term complications are related to vascular access sites [5]. Since both stent types require the same size of vascular sheath for introduction into the artery, the expected procedure-related complication rate is the same in both groups. All patients are handled as in-house patients at the Department of Vascular Surgery and will be carefully monitored according to standard team policy. Medical staff are available 24/7, and all potential complications will be handled by experienced staff.

List of potential complications:

- Death
- Arterial dissection
- Bleeding
- Contrast allergy

#### Radiation risk

CTA at 12 months has been added to validate the DUS findings. The additional radiation exposure to the patient is negligible, with approximately 2–8.6 mSv over the 5-year study period. The natural background exposure in Denmark is around 3 mSv annually [11]. The added radiation exposure of 2–8.6 mSv will increase the cancer risk in the individual patient with 0.08% providing a total lifetime cancer risk to 25.08% from 25%.

### Frequency and plans for auditing trial conduct {23}

This trial is in accordance to the local guidelines and GDPR, and Danish Good Clinical Practice (GCP) guidelines. However, during the trial period, there will be an internal monitoring of the data and included patients. In addition, the trial steering group meet monthly to review trial conduct, possible adverse events, and other matters that may need to be discussed. The trial steering committee will evaluate and validate safety and efficacy of the trial, after inclusion of 1/3 and 2/3 of subjects.

### Plans for communicating important protocol amendments to relevant parties (e.g., trial participants, ethical committees) {25}

If new information regarding effects, risks, and side effects is discovered after inclusion or during study follow-up, the patient will be informed. If the study is terminated, the study subject is to be informed of the cause of termination. If there are any protocol amendments that may impact the nature of the trial, the sponsor and funder will be notified first.

### Dissemination plans {31a}

The trial is registered at ClinicalTrials.gov. Results will be published there accordingly as well as manuscripts published in a peer-reviewed journal. All results will be published, positive or negative, and potential funding bodies will have no impact on which results are published. For publication, all data is consolidated, anonymized and individual patients and outcomes not traceable.

## Discussion

The COMESS trial will compare the use of covered stent and bare metal stent in patients with mesenteric ischemia. Treatment of mesenteric ischemia with CS is largely used in accordance with national guidelines, even though there is actually no published prospective data with this type of stent treatment. There is currently no level 1 evidence in terms of treatment of CMI and AoCMI with BMS. Hence, this trial is of great importance in terms of determining the accurate stent treatment in CMI patients. Both short and long term, patency, follow-up, QoL, and cost effectiveness are evaluated.

Research in this area is highly dynamic and the development rapid. With the current project, we can make a significant contribution to this development based on new applications of research material and the latest analysis modalities for research.

## Trial status

Patient enrolment commenced on June 1, 2023. Recruitment period is anticipated to take between 18 and 24 months. The trial is expected to complete inclusion in June 2025. Protocol version: Stent versus Covered stent treatment for chronic mesenteric ischemia__version 1.4 20,220,718 final.

## Data Availability

Research results belong to the trial site, and results are not handed out. The protocol is performed according to the Declaration of Helsinki II, and trial participants may withdraw from the trial at any time without further explanation. Any data required to support the protocol can be supplied on request.

## Abbreviations

SMA: Superior mesenteric artery
PTA: Percutaneous transluminal angioplasty
CMI: Chronic mesenteric ischemia
AoCMI: Acute on chronic mesenteric ischemia
AMI: Acute mesenteric ischemia
IMA: Inferior mesenteric artery
CA: Coeliac artery
DUS: Duplex ultrasound
CS: Covered stent
BMS: Bare metal stent
QoL: Quality of life
SOC: Standard of care
CTA: Computed tomography angiography
